# Recent Progress of Chemical Reactions Induced by Contact Electrification

**DOI:** 10.3390/molecules30030584

**Published:** 2025-01-27

**Authors:** Xinyi Huo, Shaoxin Li, Bing Sun, Zhong Lin Wang, Di Wei

**Affiliations:** 1 Beijing Institute of Nanoenergy and Nanosystems, Chinese Academy of Sciences, Beijing 101400, China; 18714526124@163.com (X.H.); lishaoxin@binn.cas.cn (S.L.); 2 School of Science, China University of Geosciences, Beijing 100083, China; sunbing@cugb.edu.cn; 3 School of Nanoscience and Engineering, University of Chinese Academy of Sciences, Beijing 100049, China; 4 Centre for Photonic Devices and Sensors, University of Cambridge, 9 JJ Thomson Avenue, Cambridge CB3 0FA, UK

**Keywords:** contact electrification (CE), solid–solid CE, solid–liquid CE, CE-Chemistry

## Abstract

Contact electrification (CE) spans from atomic to macroscopic scales, facilitating charge transfer between materials upon contact. This interfacial charge exchange, occurring in solid–solid (S–S) or solid–liquid (S–L) systems, initiates radical generation and chemical reactions, collectively termed contact-electro-chemistry (CE-Chemistry). As an emerging platform for green chemistry, CE-Chemistry facilitates redox, luminescent, synthetic, and catalytic reactions without the need for external power sources as in traditional electrochemistry with noble metal catalysts, significantly reducing energy consumption and environmental impact. Despite its broad applicability, the mechanistic understanding of CE-Chemistry remains incomplete. In S–S systems, CE-Chemistry is primarily driven by surface charges, whether electrons, ions, or radicals, on charged solid interfaces. However, a comprehensive theoretical framework is yet to be established. While S–S CE offers a promising platform for exploring the interplay between chemical reactions and triboelectric charge via surface charge modulation, it faces significant challenges in achieving scalability and optimizing chemical efficiency. In contrast, S–L CE-Chemistry focuses on interfacial electron transfer as a critical step in radical generation and subsequent reactions. This approach is notably versatile, enabling bulk-phase reactions in solutions and offering the flexibility to choose various solvents and/or dielectrics to optimize reaction pathways, such as the degradation of organic pollutants and polymerization, etc. The formation of an interfacial electrical double layer (EDL), driven by surface ion adsorption following electron transfer, plays a pivotal role in CE-Chemical processes within aqueous S–L systems. However, the EDL can exert a screening effect on further electron transfer, thereby inhibiting reaction progress. A comprehensive understanding and optimization of charge transfer mechanisms are pivotal for elucidating reaction pathways and enabling precise control over CE-Chemical processes. As the foundation of CE-Chemistry, charge transfer underpins the development of energy-efficient and environmentally sustainable methodologies, holding transformative potential for advancing green innovation. This review consolidates recent advancements, systematically classifying progress based on interfacial configurations in S–S and S–L systems and the underlying charge transfer dynamics. To unlock the full potential of CE-Chemistry, future research should prioritize the strategic tuning of material electronegativity, the engineering of sophisticated surface architectures, and the enhancement of charge transport mechanisms, paving the way for sustainable chemical innovations.

## 1. Introduction

Contact electrification (CE), a phenomenon characterized by the generation of triboelectric charges [1] at the interface between contacting materials, has been recognized for over 2600 years since its discovery [2,3]. CE has driven advancements in various technologies, including inkjet printers [4,5] and electrostatic spraying [6,7]. Despite its historical significance, the fundamental mechanisms [8] underlying CE remain incompletely understood. Initially regarded solely as a source of harm, CE-generated electrostatic charges were notorious for causing severe industrial hazards [9], including damage to electronic systems [10], explosions in coal mines [11], and fires [12]. However, in recent decades, CE has been harnessed for energy harvesting [13,14] through triboelectric nanogenerators (TENGs) [15]. By coupling CE with electrostatic induction, TENGs convert mechanical motion into electricity [16], powering devices [17] and sensors autonomously [18]. This innovation advances self-powered systems [19] and sustainable energy solutions [20], enabling efficient [21] and autonomous technologies [22,23].

CE has traditionally been regarded as a physical phenomenon. Despite its extensive history of application-oriented research [24,25,26], the atomic-scale mechanisms governing interfacial charge transfer remain an enduring challenge [27]. The concept of electron transfer is well established for metal–metal contact [28], where contact potential arises from differences in work functions [29]. For metal–insulator or insulator–insulator contacts [30], however, three primary hypotheses, electron transfer [31], ion transfer [32], and material transfer [33,34,35], have been proposed based on experimental evidence. For instance, the correlation between weakly bound ions in polymers and surface charge transfer strongly supports the role of ion migration during CE [36]. Additionally, the temperature-dependent behavior of thermal electron emissions provides compelling evidence for electron transfer in CE processes involving metals [37] and inorganic insulators [38]. These findings underscore the complexity of charge transfer mechanisms and the need for further investigation at the atomic level [39,40,41]. In addition, CE occurred even when two materials with identical chemical compositions came into contact [42]. Studies demonstrated that convex surfaces or positively curved geometries were more likely to acquire negative charges, while concave surfaces or negatively curved geometries tended to accumulate positive charges [43]. At the nanoscale, contact-charged surfaces exhibited a random “mosaic” of positively (+) and negatively (−) charged regions, resulting in a net charge with either positive or negative polarity. These charge mosaics [44] were universal in a topological sense, appearing alongside changes in surface composition and material transfer [34] between contacting surfaces. Thus, CE could not be attributed or predicted solely based on the homogeneous nature of the material. Instead, it likely arose from the chemical and micromechanical properties at and near the contacting insulator interface [45].

Studies on CE have expanded beyond the charge transfer process to explore its intriguing implications for chemical reactions. For instance, when the dielectric polymer polytetrafluoroethylene (PTFE or Teflon) [46] was repeatedly brought into contact with and separated from potassium (K) metal [47], a continuous and compact protective layer formed at the interface, effectively suppressing the growth of potassium dendrites in solid-state batteries. In another example, a charged insulating material, such as polymethylmethacrylate (PMMA) pre-treated via CE with polyethylene (PE) rubbed with Nylon, was repeatedly immersed in an inorganic metal ion solutions, including Ag_2_SO_4_, saturated PdCl_2_, or CuSO_4_ [48]. Metal precipitates adhered to the polymer surface, demonstrating the critical role of contact-induced electrostatic charges in facilitating metal reduction reactions [49,50,51]. Notably, the charge transfer at the S–L interface further amplified the chemical characteristics of the CE process. For example, Wang et al. [52] utilized the CE between fluorinated ethylene propylene (FEP) powder and water to degrade organic pollutants, introducing the concept of contact-electro-catalysis (CEC) technology. Li et al. [53] conducted an in-depth investigation of defect control and the catalytic performance of FEP by integrating experimental studies with first-principle calculations. It was found that functional group modifications influence the electronic properties of FEP. Reactive ion etching treatment effectively passivates its defects, enhancing its CE capability and CEC, thereby optimizing the overall CEC process. Furthermore, Su et al. [54] demonstrated that ultrasound-induced CEC technology drives redox reactions at the water–solid interface, effectively reducing multiple noble metal ions under both anaerobic and aerobic conditions. Wei et al. [55] expanded the range of chemical reactions induced by CE, encompassing redox, synthesis, fluorescence, and other reactions, while also introducing the concept of CE-Chemistry. CE-Chemistry harnesses the electron transfer generated by the CE effect at S–L or even liquid–liquid (L–L) interfaces to generate radicals and drive chemical reactions. In contrast to traditional catalytic strategies, CE-Chemistry is induced by external mechanical stimuli, and the catalytic materials can be chemically inert and insulting dielectrics, such as FEP [56,57], Teflon, polyvinyl chloride (PVC), polyethylene (PE), highly hydrophobic ceramics, etc. This catalytic approach opens a new pathway for non-metallic catalysis, offering broad applicability, an expanded reaction scope, and reduced secondary environmental pollution, demonstrating significant potential for advancing carbon neutrality and sustainable development.

**Figure 1:**
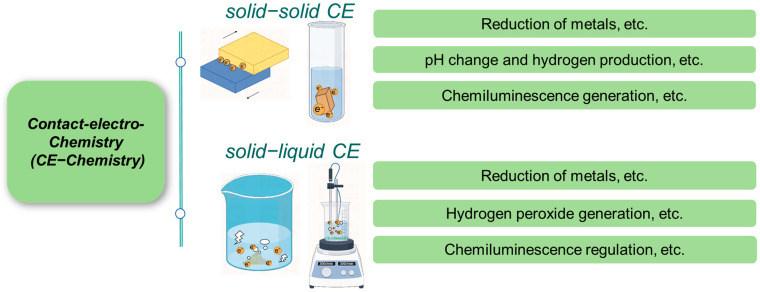
CE-Chemistry in S–S and S–L CE.

The phenomenon of CE occurs at S–L and S–S interfaces. This review provides a comprehensive overview of the characteristics and mechanisms of CE-Chemistry at these interfaces (Figure 1), encompassing fundamental principles, reaction types, regulatory strategies, and practical applications. The intrinsic mechanisms of CE-Chemistry at S–S and S–L interfaces were explained in detail, integrating the electron cloud model and the hybrid electrical double layer (EDL) model. Key factors influencing CE-Chemistry were identified, and both universal and tailored modification strategies to enhance reaction efficiency were summarized, providing actionable insights for optimizing CE efficiency [58,59,60]. This review explores the application of diverse interfaces in various chemical reactions, emphasizing the critical role of interfacial charge properties, specifically, the electronegativity of materials, in modulating CE-Chemistry processes. This deepens the understanding of CE’s physicochemical nature and broadens the scope of CE-Chemistry technologies. The review concludes by highlighting future opportunities and challenges in CE-Chemistry, aiming to foster ongoing development and innovation in the field.

## 2. Theoretical Model of Charge Transfer for CE

**Figure 2:**
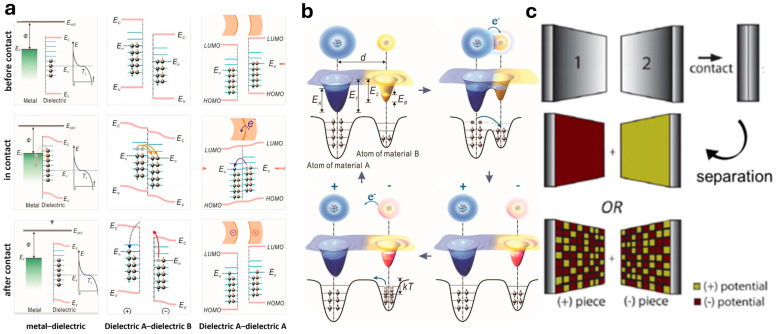
(**a**) Energy bands between a metal and a dielectric, between two different dielectrics, and between identical dielectrics during contact–separation process (reprinted with permission from Ref. [62]. 2024, Springer). (**b**) Schematic of the electron cloud and potential energy profile of two atoms belonging to two materials A and B during CE (reprinted with permission from Ref. [63]. 2022, John Wiley and Sons). (**c**) The traditional view (uniformly positively or negatively) and the mosaic picture model (positively charged and negatively charged regions on the nanoscale) during contact–separation process, respectively (reprinted with permission from Ref. [45]. 2011, American Association for the Advancement of Science).

When two solids come into contact, charge transfer occurs between their interfacial atoms or molecules. One material loses electrons, becoming positively charged, while the other gains electrons, acquiring a negative charge [61]. Numerous discussions have addressed the nature of this charge transfer, and various theoretical models have been proposed to elucidate the mechanisms of S–S CE. Figure 2a illustrates the energy band configurations for various contact scenarios: between a metal and a dielectric, between two different dielectrics, and between identical dielectrics [62]. Typically, the electrons on the surface of an insulating dielectric material occupied energy levels lower than the Fermi energy level of the metal, leaving many surface states unoccupied. When the temperature reached a certain threshold, some electrons in the metal acquired sufficient energy to surpass the Fermi energy level. These high-energy electrons transferred from the metal to the unoccupied surface states of the dielectric material upon contact [63]. Additionally, contact between two chemically identical materials (dielectric A and dielectric A) also generated electrostatic charges, with the direction of charge transfer influenced by the surface curvature of the samples. Surfaces with positive curvature (a convex surface) typically acquired a net negative charge, whereas surfaces with negative curvature (a concave surface) exhibited a net positive charge [43]. CE in dielectric–dielectric systems represented a universal phenomenon, observable across all materials, including semiconductors and dielectrics with electronic structures describable by energy bands [30]. Most of the transferred electrons on the dielectric surface remained after the materials separated, owing to the presence of energy barriers. This retention was particularly significant for chemical reactions, as it contributed to reducing the activation energy of the reactions. When the electron charge accumulates on the surface of a polymer beyond a critical threshold, the resulting high surface voltage can lead to air breakdown, which may damage the surface material [64]. Therefore, minimizing excess charge accumulation can enhance the charge stability and longevity of the dielectric. Furthermore, controlling the extent of charge transfer would allow for better modulation of interfacial interactions, thereby improving device efficiency and reliability. Wang et al. [62] proposed an electron cloud potential model to elucidate the differences in energy band structures between material A and material B, as illustrated in Figure 2b. This model was applicable to interfacial CE across various material types and emphasized charge transfer at the atomic level. In this framework, atoms were depicted as potential wells with loosely bound electrons occupying specific orbital electron clouds. Before the materials made contact, electron transfer was inhibited by the local capture effect of the potential wells. Upon contact between material A and material B, the overlap of electron clouds caused the initial single potential well to transform into an asymmetric double potential well, facilitating electron transfer from material A to material B. In most macroscopic potential measurement experiments, the surface potentials of both materials changed following contact and separation, exhibiting either positive or negative potentials. Lacks et al. [27] discovered that polymer surfaces exhibited a charge pattern consisting of nanoscale regions with alternating positive and negative charges following CE. When two polymers came into contact, their charge distribution surfaces were characterized at the nanoscale using Kelvin force microscopy [45]. It was revealed that the surface charge of dielectric materials after CE formed a random “mosaic” of positively (+) and negatively (−) charged regions, as depicted in Figure 2c, at the bottom right. These mosaics were found to be topologically universal, displaying consistent characteristic length scales across different materials. Despite holding significantly higher charge densities per unit area than previously estimated for CE, the total or “net” charge on the surface remained relatively small due to the mutual “compensation” of the opposing charge regions. The formation of these charge mosaics was further associated with alterations in surface composition and material transfer between the contacting surfaces. Baytekin et al. [45] proposed that CE was potentially influenced by the chemical and micromechanical properties of the contacting dielectric surfaces. This observation established a connection between CE and surface chemical changes, underscoring the physicochemical implications of the CE process. The theoretical model of charge transfer for CE holds the potential to revolutionize the understanding of interfacial dynamics. Its insights could inspire new approaches for optimizing material properties and advancing next-generation energy storage, catalysis, and environmental technologies.

## 3. CE-Chemistry Induced by S–S CE

**Figure 3:**
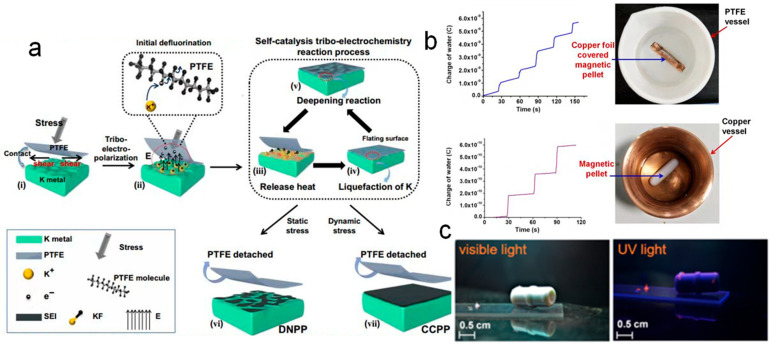
(**a**) Schematic diagram of self-catalysis tribo-electrochemistry reaction process (reprinted with permission from Ref. [47]. 2024, Springer). (**b**) The charge signal acquired by the water in a Teflon beaker and in the copper vessel, respectively. Photograph of the Teflon beaker containing 100 mL water with a magnetic pellet covered by a copper foil and the copper vessel containing 50 mL water with a magnetic Teflon pellet. (**c**) Photographs of the reaction product of Teflon and gold in the presence of glucose under visible light and UV light (mercury vapor lamp) (reprinted with permission from Ref. [70]. 2019, American Chemical Society).

Charge transfer during CE at S–S interfaces typically occurs within a very short time frame, with charge carriers easily stored on the surface of the insulating dielectric following separation [65,66]. Due to their excellent charge storage capacity, the surfaces of dielectric materials undergo repeated charge transfer and accumulation through successive contact and separation [67,68,69], exhibiting more pronounced phenomena in CE, as seen in materials such as Teflon and FEP. These materials have traditionally been considered chemically inert due to their stable properties. However, in 2019, Wang et al. [47] proposed a novel self-catalytic triboelectric charge reaction occurring between Teflon and potassium (K) metal. In this process, a continuous and compact protective layer was rapidly formed on the K metal anode within seconds, effectively reducing the growth of K dendrites. As illustrated in Figure 3a, the mechanism underlying the self-catalytic reaction includes the following stages: (i) the application of force as an initiator for charge accumulation; (ii) the electric field generated by the accumulated charge acting as a catalyst to induce initial defluorination, accompanied by heat release; (iii) heat accumulation resulting from the ongoing reaction; (iv) the liquefaction of potassium (K) metal due to the heat, enabling deeper reaction; and (v) propagation of the reaction driven by increased heat, leading to the formation of an artificial solid electrolyte interface (ASEI). Notably, if the accumulated charge surpasses a critical threshold, an “explosive” reaction occurs. Leveraging this phenomenon, continuous compact protected potassium (CCPP) anodes were successfully prepared, demonstrating enhanced K^+^ diffusion kinetics, improved ionic conductivity, reduced electronic conductivity, and effective suppression of potassium dendrite formation. This study underscored the significant role of CE in driving chemical reactions, even in interactions involving chemically inert polymeric materials. The charge transfer facilitated by S–S CE proved pivotal in forming a protective film at the interface, effectively mitigating dendrite growth.

Furthermore, similar CE-induced reactions between inert polymers and metals have been observed in solution-phase conditions. For instance, Nag et al. [70] reported the degradation of Teflon in water in the presence of common metals and carbohydrates, producing polymer fragments. Teflon particles were stirred with glucose in water at 70 °C, resulting in the isolation of a solid material composed of polymer fragments and metallic copper from a copper vessel after 15 days. The formation of fluorocarbons in the solution was confirmed through high-resolution electrospray ionization mass spectrometry (HREIMS). This process involved CE at the Teflon surface during stirring and the subsequent interaction of the charged surface with metal ions, as depicted in Figure 3b. This phenomenon was found to extend to other polymers, such as FEP. Notably, when gold (Au) ions reacted with Teflon in the presence of glucose through CE, the resulting solid exhibited a red fluorescence under UV light, a characteristic not observed with other metals, as shown in Figure 3c.

**Figure 4:**
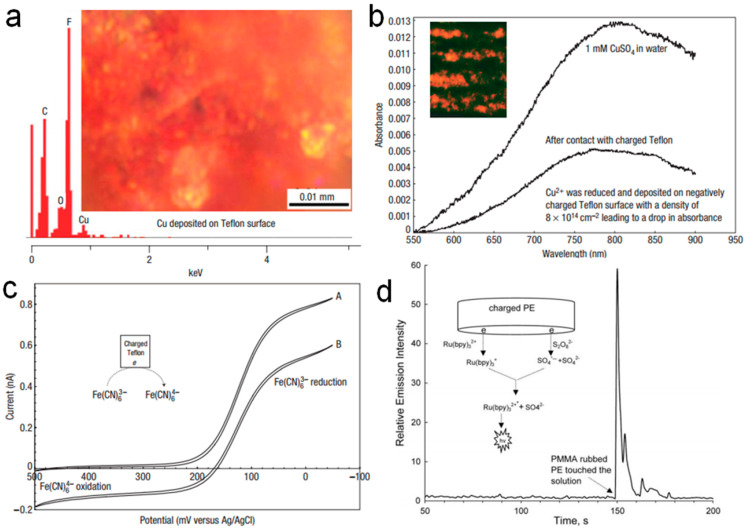
(**a**) EDS of Cu deposited on charged Teflon. (**b**) Optical absorbance of 1 mM CuSO_4_ solution before and after contact with charged Teflon. (**c**) Fe(CN)_6_^3−^ was reduced by charged Teflon to Fe(CN)_6_^4−^ (reprinted with permission from Ref. [71]. 2008, Springer Nature). (**d**) Relative intensity of chemiluminescence as a function of time when a PMMA-rubbed PE rod dipped into a MeCN/H_2_O (1:1, *v*/*v*) mixture containing 2.5 mM S_2_O_8_^2−^ and 0.25 mM Ru(bpy)_3_^2+^. Insert shows the reaction mechanism (reprinted with permission from Ref. [74]. 2009, Elsevier).

In 2008, Liu et al. [71] utilized Faraday’s law to determine charge density and demonstrated the pivotal role of electron transfer in chemical reactions mediated by charged polymers through CE. They employed charged Teflon as a single electrode in a solution to drive various chemical reactions, including pH alterations, hydrogen generation [72,73], and metal reduction. For instance, after rubbing Teflon with Plexiglas, the charged Teflon was immersed in a CuSO_4_ solution, triggering the reaction: Cu^2+^ + 2e^−^ → Cu. Energy dispersive X-ray spectroscopy (EDS) analysis of the Cu film (Figure 4a) confirmed the deposition of metallic Cu on the Teflon surface. EDS scans consistently revealed Cu peaks at all examined spots, while no Cu was detected on uncharged Teflon. The observed F and C peaks in Figure 4a originated from the underlying Teflon layer beneath the Cu spots, which was sufficiently thin to allow for penetration of the electron beam. Notably, the F-to-C peak ratio in the EDS scans was significantly higher for bare Teflon compared to Cu-deposited regions, suggesting a potential depletion of F atoms beneath the Cu spots. Moreover, Figure 4b shows the absorbance comparison of the CuSO_4_ solution before and after contact with charged Teflon, substantiating the reduction of copper ions by the charged surface. The change in concentration after contact with Teflon corresponded to an average surface charge density of approximately 8 × 10^14^ cm^−2^ (based on the geometric area). This value, while slightly lower, remains the same order of magnitude as the charge density determined from the observed pH change. Similarly, in the reduction of Fe(CN)_6_^3−^, uncharged Teflon immersed in a Fe(CN)_6_^3−^ solution showed no evidence of Fe(CN)_6_^4−^ formation after separation. However, charged Teflon facilitated the reduction of Fe(CN)_6_^3−^ to Fe(CN)_6_^4−^. This was evident in Figure 4c, where cyclic voltammetry displayed a steady-state current plateau for Fe(CN)_6_^3−^ in the initial solution. After contact with charged Teflon, the plateau height decreased, and a new anodic plateau corresponding to Fe(CN)_6_^4−^ emerged, confirming the reduction reaction. Additionally, Figure 4d illustrated the relative intensity of chemiluminescence over time as polymethylmethacrylate (PMMA) charged by CE with a polyethylene (PE) rod was immersed in a MeCN/H_2_O mixture containing S_2_O_8_^2−^ and Ru(bpy)_3_^2+^. A sharp increase in fluorescence intensity occurred upon the entry of charged PE rods, signifying that the fluorescence enhancement originated from the surface charge of the PE material. This study holds significant potential for microelectronic and related applications, given Teflon’s advantageous properties, such as a low dielectric constant and excellent thermal stability. By employing Faraday’s law, the charge density was precisely quantified, underscoring the critical role of electron transfer processes on charged polymers by CE and potentially extending to other insulating materials.

**Figure 5:**
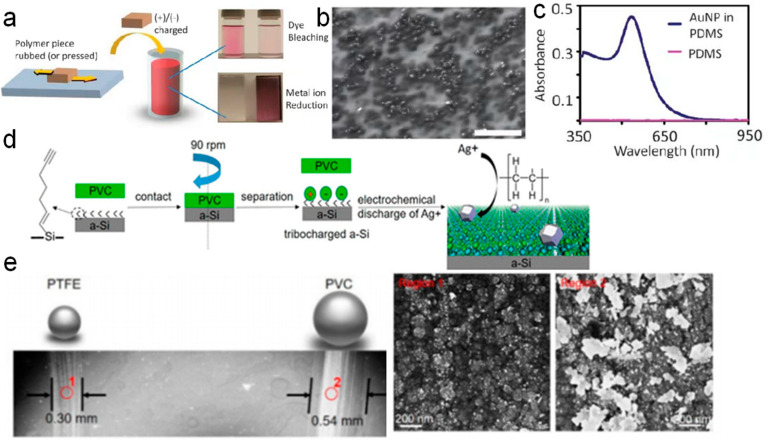
(**a**) Polymer pieces are charged by rubbing or pressing against one another and are then immersed in an aqueous reagent solution. (**b**) SEM image zooming on the Au NPs deposited on the surface of (+) contact-charged PDMS (scale bar = 1 μm). (**c**) UV−vis spectrum featuring a surface plasmon resonance (SPR) peak centered at 520 nm and characteristic of Au nanoparticles (reprinted with permission from Ref. [75]. 2012, American Chemical Society). (**d**) Schematic for the electrodeposition of silver nanoparticles on tribocharged undoped amorphous silicon (a-Si) samples. (**e**) SEM images of patterns of silver particles generated by reducing silver ions on a-Si samples tribocharged by rolling PVC and Teflon spheres (reprinted with permission from Ref. [33]. 2021, American Chemical Society).

Some findings suggested that the initiation of chemical reactions via CE in chemically inert dielectric materials was due to the presence of surplus “cryptoelectrons” on the material’s surface. These electrons were subsequently transferred to the solution, where they interacted with reactants to trigger chemical reactions. Alternatively, Baytekin Bilge et al. [45] proposed that the CE effect was driven by free radicals generated during solid dielectric–solid dielectric contact. They observed that CE induced the formation of positively and negatively charged regions of the nanometer scale on both contacting surfaces. Notably, they proposed that both surfaces, rather than just the negatively charged one, could provide “cryptoelectrons” and participate chemically. Consequently, polymer-surface-driven reactions were influenced not only by electrical factors but also by mechanically generated radicals, with these processes accompanied by surface charging and material transfer during contact or separation [75]. To validate this hypothesis, they employed various polymers to test CE by pressing them together and separating them or by mechanically rubbing them and subsequently using the contact-charged surfaces to reduce metal salts and bleach redox-reactive dyes. As illustrated in Figure 5a, polymer fragments underwent CE through rubbing or pressing, resulting in surface charging. The charged fragments were then immersed in reactive solutions, where it was observed that both negatively and positively charged regions facilitated chemical reactions in the solution. For instance, by extruding polymers such as polydimethylsiloxane (PDMS), PMMA, Teflon, PVC, polystyrene (PS), polycarbonate (PC), and polyoxymethylene (POM) and subjecting them to contact charging, either through pressing and subsequent separation or mechanical rubbing, the neutral red dye was observed to bleach upon contact with the positively charged surfaces of all the aforementioned polymer sheets [75]. Similarly, PDMS was positively charged through friction with Teflon, and PDMS enabled the reduction of HAuCl_4_(aq) into Au nanoparticles [75]. The SEM images of Au nanoparticles deposited on the PDMS surface are shown in Figure 5b, and the UV–vis spectra of the nanoparticles are presented in Figure 5c.

The authors further explored the reduction of pH-dependent dye indicators by charged polymers. If the observed dye bleaching resulted from electron-mediated reduction, the extent of this reduction would be expected to increase with decreasing pH. However, the experimental results contradicted this expectation, showing that bleaching increased with rising pH for both Neutral Red (NR) and Methylene Blue (MB) [33] aqueous solutions. Moreover, when mixtures of these dyes were treated with charged polymers, their bleaching behavior did not align with predictions based on standard electrode potentials for a redox process. Instead, the order of bleaching was opposite that of the anticipated sequence. Based on these findings, Baytekin et al. [75] proposed that the mechanical deformation of dielectric polymers invariably induces charge transfer during CE or friction, which in turn drives chemical reactions. They argued that electrons are not the primary agents in the CE reaction; rather, mechanical stress generates free radicals within the polymer. These radicals, not electrons, are responsible for the observed reactivity. The presence of such free radicals on the surface of polymers charged through mechanical contact was confirmed using magnetic force microscopy [75]. Furthermore, Zhang et al. [33] investigated how electrostatic charges on insulating dielectric surfaces could be harnessed to enhance chemical reactivity. They proposed leveraging the CE effect to enable localized frictional electrification as a novel approach for redox lithography. An example of surface redox reactivity induced by CE was demonstrated between an organic monolayer-modified silicon wafer and PVC. The process of electrodepositing silver nanoparticles on a tribocharged undoped amorphous silicon (a-Si) sample was schematically illustrated in Figure 5d, highlighting the correlation between surface adhesion and the accumulation of electrostatic charge on the insulator in CE-induced chemical reactions. As shown in Figure 5e, an SEM image depicted the silver particle pattern generated by rolling PVC and Teflon spheres on an a-Si sample, followed by immersing the tribocharged sample in an aqueous AgNO_3_ solution to reduce silver ions [33]. A marked variation in the density and size of the silver particles was observed, underscoring the influence of CE on the resulting chemical reactivity and particle formation.

**Figure 6:**
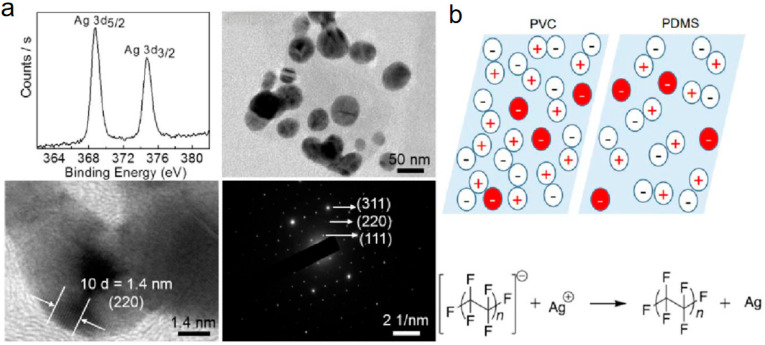
(**a**) Analysis of silver nanoparticles electrochemically grown on electrical insulators (tribocharged PDMS samples immersed in aqueous AgNO_3_ solutions). (**b**) Schematic depiction of a “mosaic” ensemble of triboelectric charges on two samples of equal charge excess (reprinted with permission from Ref. [76]. 2019, American Chemical Society).

Zhang et al. [33] further proposed that chemical reactions initiated by the CE of polymers are governed more by the stability of the surface charge than by the magnitude of the charge itself. They also noted that the maximum extent of redox reactions is inherently dependent on the properties of the material. The formation of silver nanoparticles on triboelectric-charged PDMS samples immersed in aqueous AgNO_3_ was analyzed, as shown in Figure 6a. Transmission Electron Microscopy (TEM) revealed silver particles with diameters of approximately 50 nm, while High-Resolution Transmission Electron Microscopy (HRTEM) showed a lattice spacing of 0.14 nm, corresponding to the (220) planes of face-centered cubic (fcc) Ag [76]. The schematic in Figure 6b illustrates the triboelectric charge distribution on two equally charged samples, highlighting how variations in ionization energies affect charge domain aggregation. For instance, although the net charge densities of PVC and PDMS samples were equal (red symbols), the differences in their negatively charged domains were significant. Debris from Teflon following CE was found to mediate the reduction of Ag^+^ ions. This reduction led to the nucleation of silver particles on charged plastic surfaces. These observations suggest that polymer fragments act as mediators in redox reactions, playing a critical role in particle nucleation and growth. This study provided evidence of a material-specific slope in the redox work versus static charge density curves, emphasizing that a sensitivity factor needed to be considered when using plastics to deliver a precise quantity of charge, measured in coulombs, to the reaction mixture. Materials with large negative electron affinities (EA) and relatively low ionization energies (IE), stabilizing cations while destabilizing anions, and mediated redox work to a considerable extent. Conversely, dielectric materials with stable anions (less negative EA) and large IE were likely to be more effective for delivering small redox changes with high precision. Collectively, these findings constituted the most compelling evidence to date that the triboelectric charge in polymers might be ionic species. This work broadened the understanding of electrostatic electricity and identified potential applications in single-electrode electrochemistry and the exploration of electrostatic catalysis in chemical reactivity. CE-Chemistry induced by S–S CE offered groundbreaking possibilities for controlling chemical reactions at the interface, potentially leading to the design of more efficient, environmentally friendly processes. This approach could unlock new frontiers in catalysis, energy conversion, and environmental remediation, establishing a foundation for future research in sustainable electrochemical technologies.

## 4. Chemical Reactions Induced by S–L CE

### 4.1. S–L CE

**Figure 7:**
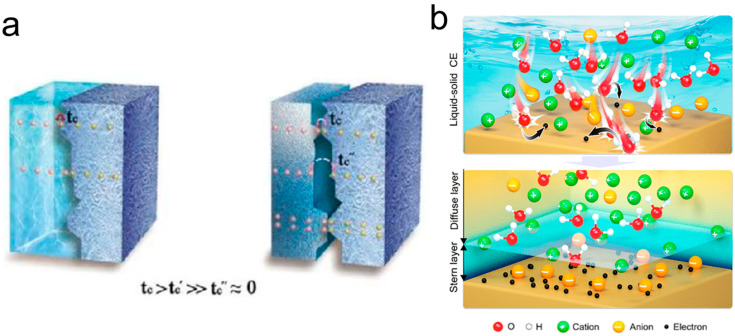
(**a**) Schematic diagram of a unified mechanical model of contact charge charges (reprinted with permission from Ref. [86]. 2020, John Wiley and Sons). (**b**) Lin’s hybrid EDL model and the “two-step” process on its formation. In the first step, the molecules and ions in the liquid impact the solid surface due to the thermal motion and the pressure from the liquid, which leads to electron transfer between them; meanwhile, ions may also attach to the solid surface. In the second step, free ions in the liquid would be attracted to the electrified surface due to electrostatic interactions, forming an EDL (reprinted with permission from Ref. [87]. 2022, American Chemical Society).

CE ubiquitously occurs at interfaces. Beyond the well-studied S–S interface, the phenomenon of CE at the S–L interface has garnered increasing attention [59,77]. Historically, research on CE predominantly focused on S–S interactions, leaving S–L CE relatively unexplored [78,79,80]. In the 1980s, EI-Kazzaz and Rose-Innes [81] investigated CE between liquid metals and solid insulators, marking the early exploration of S–L CE. By leveraging the fluidity of liquid metals to improve the interface contact area, they sought to advance the understanding of S–S CE mechanisms but did not fully elucidate S–L CE processes. In the 1990s, Matsui and Yatsuzuka et al. [82] examined the CE behavior between water droplets and insulating surfaces, discovering that water droplets consistently acquired a positive charge when sliding on insulator surfaces such as Teflon and resin. More recently, in 2016, Burgo et al. [83] quantified the position of water within the triboelectric series. These studies attributed the electrification of water upon contact with insulators to the adsorption of negative ions on the insulator surface; however, conclusive evidence supporting this hypothesis remained lacking. In recent years, numerous studies have redirected attention toward the properties of charge carriers in S–L CE, exploring the phenomenon at both the nanoscale and macroscale [46,84]. These investigations have demonstrated the coexistence of electron and ion transfer in S–L CE, with electron transfer occasionally playing a dominant role. Distinguishing between ionic and electronic charge transfer can be achieved through two methodologies: temperature-induced electron-thermo-ion emission [63] and UV-induced electron emission [85]. Notably, at moderate temperatures, neither method releases surface-adsorbed ions, indicating that thermal excitation can stimulate the generation of surface charges in S–L CE, thereby providing direct evidence of electron transfer in such systems [84]. Additionally, computational studies have revealed that the ion concentration in deionized water is insufficient to account for the observed charge densities in S–L CE, further substantiating the role of electron transfer [46]. Morten Willatzen et al. [86] proposed a unified quantum mechanical model of CE that can represent metals, semiconductors, or insulators in fluid or solid phases with effective electron transfer parameters such as the driving mechanism, as shown in Figure 7a. This model is well consistent with the known experimental results, such as the charging of similar materials, surface charge mosaicking, and higher charge transfer efficiency in S–S contact compared to S–L contact, and found that Coulomb interactions affect not only the transfer of the charge but also the period of oscillation of the charge.

Lin et al. [87] extended the understanding of CE at the S–L interface by considering both electron transfer and ion adsorption phenomena. They proposed a model for the formation of EDLs during the S–L CE process. As illustrated in Figure 7b, the process begins with molecules and ions in the liquid colliding with the solid surface due to thermal motion and liquid pressure. The overlap of electron clouds between solid atoms and water molecules induces electron transfer. For instance, solid dielectric materials with strong electron-capturing abilities, such as fluorine-rich polymers, can directly capture electrons from water molecules or even ions in the liquid. Subsequently, adjacent liquid molecules near the solid surface are displaced from the interface by liquid flow or turbulence, further contributing to the EDL formation. The electron transfer process in S–L CE is governed by the transition of electrons from a high-energy to a lower-energy state. Following separation, most transferred electrons remain on the solid surface. In the subsequent step, free ions in the liquid are electrostatically attracted to the charged surface, leading to the formation of an EDL analogous to conventional EDL models. Concurrently, ionization reactions occur at the solid surface, producing both electrons and ions. For instance, water molecules losing electrons become cations [88], triggering the reaction [89]: H_2_O^+^ + H_2_O→·OH + H_3_O^+^. This reaction generates hydroxyl radicals (·OH) and hydronium ions (H_3_O^+^). The disrupted water molecules displaced from the solid surface transform into free-migrating ions within the liquid, further contributing to EDL formation. This two-step model offers a novel perspective on EDL formation, with significant implications for foundational chemistry and mechanochemistry.

### 4.2. Chemical Reactions Induced by S–L CE

**Figure 8:**
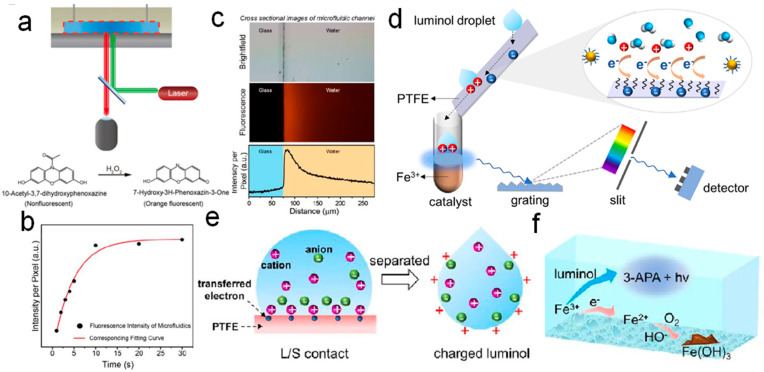
(**a**) Schematic image of fluorescence microscopy set up for imaging the microfluidic chip. (**b**) The relationship between fluorescence intensity and reaction time of sample resting in microfluidic chip. (**c**) The fluorescent profiles of the spontaneous generation of H_2_O_2_ along the normal direction of the substrate (top, the optical microscopy image of a typical straight channel; middle, the corresponding fluorescence image; bottom, the corresponding fluorescence intensity) (reprinted with permission from Ref. [90]. 2022, American Chemical Society). (**d**) When a drop of luminol solution flowed through the Teflon surface, the electron transfer between luminol and solid occurred, and then the statically charged luminol was dropped into the cuvette containing the catalyst, leading to light blue emission. (**e**) Schematic depiction of the electron transfer between a liquid droplet and the Teflon interface and the ions adsorbed due to the Coulombic attraction. After separation, the positively charged luminol droplet attracted anions at the interface. (**f**) Schematic depiction of the electrons from the negatively charged luminol droplet competing with luminol for Fe^3+^ (reprinted with permission from Ref. [91]. 2022, American Chemical Society).

Beyond the investigation of CE properties between water and solid dielectric materials, the physicochemical processes underlying this interaction have garnered significant interest. For instance, Chen et al. [90] demonstrated that H_2_O_2_ is spontaneously generated through the formation of hydroxyl groups at S–L interfaces during contact. The density of these hydroxyl groups was shown to directly influence the yield of H_2_O_2_. This conclusion was substantiated through mass spectrometry and complementary analytical techniques, providing robust evidence for the critical role of ·OH radicals in the generation of H_2_O_2_. Chen et al. [90] constructed an idealized water–solid interface by sealing patterned PDMS plates onto flat glass substrates, creating a typical straight-through microfluidic chip (Figure 8a). This design effectively eliminated the influence of gas-phase substances, isolating the spontaneous generation of H_2_O_2_ at the S–L interface. The production of H_2_O_2_ within the microfluidic chip was quantified using a water-soluble probe sensitive to H_2_O_2_. Fluorescence microscopy revealed the relationship between the S–L interface and fluorescence intensity (Figure 8b). A fluorescence profile captured the distribution of spontaneously generated H_2_O_2_ along the axis perpendicular to the substrate, with corresponding optical microscopy, fluorescence images, and intensity profiles, shown from top to bottom in Figure 8c. The study [91] introduced a novel perspective on water–solid interactions, providing critical insights into the role of S–L CE in chemical processes. The findings align with conventional electrochemistry, emphasizing the importance of electron transfer in directing reaction pathways, a principle equally crucial in CE-Chemistry. In conventional electrochemistry, electron transfer plays a pivotal role in dictating the course of chemical reactions, a principle similarly observed in CE-Chemistry. Zhang et al. [91] explored the modulation of chemiluminescence via electron transfer at the S–L interface. They began by analyzing electrostatic interactions between electrostatically charged luminol droplets and a directional static field, utilizing a droplet TENG to establish the relationship between droplet polarity and its impact on reaction dynamics. Their findings demonstrated that when a luminol droplet flowed over a Teflon surface, the strong electronegativity of Teflon induced CE, causing the droplet to acquire a positive charge. Upon entering a cuvette containing a catalyst, the electrostatically charged droplet triggered an enhanced chemiluminescence reaction, emitting a vivid blue light. This increase in luminescence intensity was directly correlated with improved chemiluminescence reactivity (Figure 8d). These results provide new insights into the interplay of charge polarity, electron transfer, and reaction efficiency in CE-Chemistry. However, negatively charged droplets were found to inhibit chemiluminescence (CL), providing direct evidence that the tribocharged carriers at the S–L interface are electrons. The mechanism of CE regulation by electron transfer at the S–L interface is illustrated in Figure 8e, where a schematic representation depicts electron transfer between the droplet and the Teflon interface, along with the adsorption of ions due to Coulombic attraction. Zhang et al. [91] also explored the potential for anion aggregation at the interface of the luminol droplet due to Coulombic forces, which increased the concentration of hydroxide ions (OH^−^) at the interface. When the luminol droplet interacted with a Fe^3+^ solution, the higher concentration of OH^−^ ions enhanced CL. However, it is important to note that electrons from the self-contained negatively charged luminol droplets could compete with the luminol molecules for Fe^3+^, thereby inhibiting CL. The transfer of electrons from a negatively charged droplet is schematically shown in Figure 8f. This study extended our understanding of electrostatically induced reactions by clarifying the electrostatic regulation of CE. It suggested that electron transfer at the liquid–solid interface plays a more critical role than material transfer, ion transfer, or surface charge stability. Furthermore, it provided a strategy to promote or inhibit CE-Chemistry simply by reacting to the solution with a solid dielectric electrostatic charge, thus offering valuable insights for the manipulation of electrostatic reactions in chemistry.

**Figure 9:**
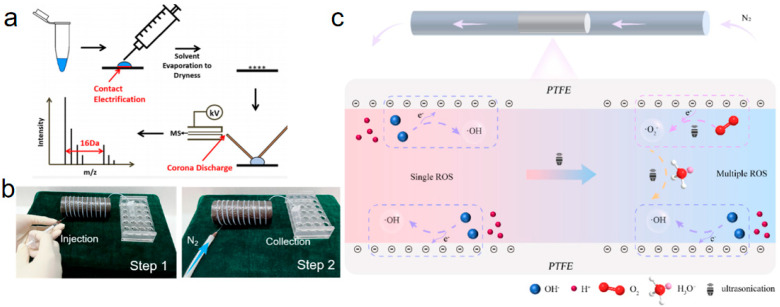
(**a**) Charge-induced peptide oxidation by S–L contact. (**b**) Photographs of the experimental setup (reprinted with permission from Ref. [93]. 2023, American Chemical Society). (**c**) Mechanism of free radical generation by CE process (reprinted with permission from Ref. [94]. 2023, Elsevier).

During S–L CE, the electron clouds of the solid and liquid surfaces overlapped, facilitating electron transfer at the interface. This interaction induced a redistribution of ions in the solution due to electrostatic interactions with the charged solid surface [30]. The involvement of CE in chemical reactions was increasingly corroborated by researchers. For instance, Wang et al. [92] utilized a custom liquid micro-junction surface sampling probe (LMJ-SSP) platform to observe reproducible peptide oxidations. Their findings revealed that factors such as the evaporation time of the sample droplet, the frequency of drying, the water content within the droplet, and the nature of the solid surface material significantly influenced the degree of analyte oxidation, thereby validating the pivotal role of S–L CE in this process. To mitigate the unwanted oxidation of analytes, they recommended reducing the water content in the sample solution and avoiding hydroxyl-functionalized substrates, such as glass carriers, as depicted in Figure 9a. This work underscored the importance of controlling interfacial conditions to manage the chemical outcomes mediated by S–L CE. Furthermore, when water served as a necessary solvent component, the addition of antioxidants, such as ascorbic acid, to the sample solution prior to droplet evaporation on the solid surface effectively reduced the extent of analyte oxidation. This approach was applicable across all mass spectrometry methods involving the drying of microliter-scale sample solutions onto substrates during sample preparation protocols. In parallel, Zhao et al. [93] observed that ·OH was generated when a droplet came into contact with a solid in the absence of external interferences. Their findings indicated that the concentration of ·OH increased with pH, implying that ·OH originated from OH^−^ ions via electron transfer during CE. The experimental setup, depicted in Figure 9b, involved injecting a water droplet into a capillary tube using a syringe, propelling it through a long capillary tube with a nitrogen gas stream, and subsequently collecting the liquid at the tube’s outlet for free radical detection. This work highlighted the role of CE-driven electron transfer in radical generation and its dependence on pH conditions. Figure 9c schematically illustrates the principle of free radical generation via CE. Electron transfer from the liquid to the solid was driven by the lower energy state of the solid and was facilitated by ultrasonication, which provided the energy necessary for electron release. Initially, complete S–L contact occurred between the liquid and the capillary wall. Due to Teflon’s higher electronegativity compared to water, electrons transferred from OH^−^ to the Teflon surface during contact. This process [93] oxidized OH^−^ ions to form ·OH, while the Teflon surface gained electrons. Simultaneously, free hydrogen ions combined with water molecules to form hydrated hydrogen ions, some of which were drawn to the Teflon surface through electrostatic interactions. Under ultrasonic excitation, oxygen molecules released into the system received electrons from the Teflon surface and were reduced to superoxide radicals (·O_2_^−^). These superoxide radicals subsequently participated in chain reactions with free water and hydrogen ions, producing additional ·OH radicals. In summary, CE involving weak mechanical forces primarily generated ·OH radicals. However, under strong mechanical forces, such as those induced by ultrasonication, CE generated both ·OH and ·O_2_^−^ radicals, expanding its chemical reactivity.

**Figure 10:**
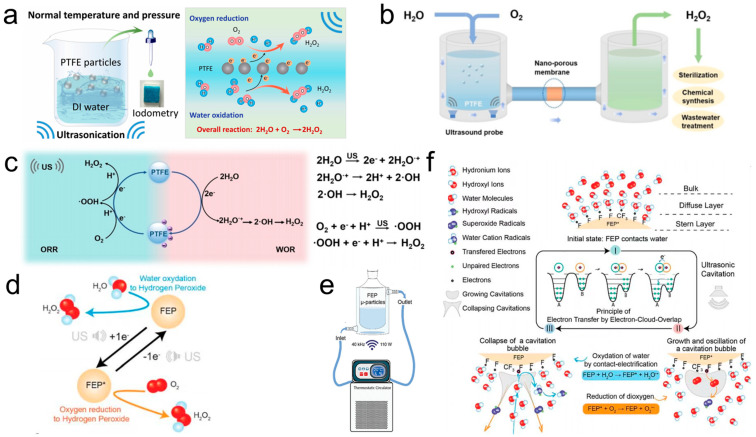
(**a**) Schematic representation of the experimental setup and overall reaction. (**b**) Expected large application of S–L CE in the field of efficient production of H_2_O_2_. (**c**) Proposed reaction mechanism of CEC for H_2_O_2_ generation (reprinted with permission from Ref. [71]. 2023, John Wiley and Sons). (**d**) Schematic illustration of generating H_2_O_2_ from water and H_2_O by ultrasonication in the presence of FEP. (**e**) Illustration of the experimental setup comprising a thermostatic circulator that regulates the temperature of the reactor and an ultrasonic bath (40 kHz, 110 W). (**f**) Illustration of the proposed general principle of the mechanism of radicals’ generation by ultrasonication-induced CEC in aqueous solution (reprinted with permission from Ref. [73]. 2023, John Wiley and Sons).

The performance of CE was found to be closely linked to the strength of external mechanical forces, with strong mechanical forces enhancing charge transfer efficiency at the interface. Zhao et al. [72] utilized ultrasonic mechanical forces to promote electron transfer at the S–L interface, enabling the synthesis of H_2_O_2_. The experimental setup and overall reaction schematic are illustrated in Figure 10a. Polymer solid dielectric materials used in CE-Chemistry were highlighted as cost-effective and readily accessible. As depicted in Figure 10b, ultrasonic mechanical force facilitated electron transfer at the S–L interface, enabling H_2_O_2_ synthesis [72]. This synthesis occurred via a catalytic pathway at room temperature and atmospheric pressure. Under mechanical force, the Teflon–water and Teflon–O_2_ interfaces underwent electron transfer upon contact, producing reactive radicals. These radicals subsequently reacted to form H_2_O_2_, as shown in the schematic diagram of the reaction mechanism in Figure 10c. This system demonstrated potential for achieving long-term, stable, large-scale production of H_2_O_2_, paving the way for the commercialization of contact electrocatalytic H_2_O_2_ synthesis. This work introduced a new approach for the efficient preparation of H_2_O_2_ and provided a foundation for further exploration of contact charge-induced chemical processes.

Similarly, Berbille et al. [73] demonstrated the use of ultrasonication to induce S–L CE for initiating an oxidation reaction, as illustrated in Figure 10d. FEP was employed as a catalyst to synthesize H_2_O_2_. The experimental setup, depicted in Figure 10e, incorporated a thermostatic circulator to regulate the reactor temperature and an ultrasonic bath. The general mechanism of ultrasound-induced free radical generation via CEC in an aqueous solution is shown in Figure 10f. Initially, when the polymer solid powder came into contact with water, CE caused the solid to acquire electrons from the liquid. During this process, a small fraction of water was oxidized to water radical cations (H_2_O·^+^), subsequently forming ·OH. Upon fresh contact, ions accumulated on the charged particle surfaces, creating a static EDL. Concurrently, the dissolved O_2_ in water formed bubbles under ultrasonication. The O_2_ within these bubbles gained electrons from the surface of the Teflon particles, forming ·O_2_^−^. These radicals reacted with hydronium ions (H_3_O^+^) to produce hydroperoxyl radicals (HO_2_·), which then interacted with H^+^ to form peroxyl radicals (·OOH). Subsequently, these radicals further gained electrons and combined with H^+^ to form hydroperoxyl radicals (HO_2_·), which ultimately led to the formation of ·OH and, through further reactions, H_2_O_2_. This work underscored the effectiveness of ultrasonication in driving S–L CE-induced chemical processes, particularly for the sustainable synthesis of H_2_O_2_.

**Figure 11:**
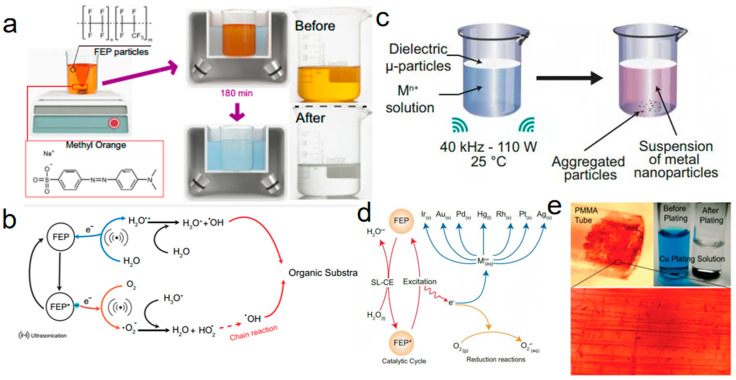
(**a**) Three-dimensional schematic of the experimental setup and protocol. (**b**) Proposed mechanism for the degradation of MO by CEC-generated radicals. (**c**) Graphical description of the experimental set-up and observations. (**d**) Schematic description of the reduction of various metal ions in aqueous solution by ultrasonically driven CEC in presence of FEP (reprinted with permission from Ref. [54]. 2024, Elsevier). (**e**) Copper plated on untreated PMMA surface. (Upper left) An optical image of copper film deposited on a PMMA tube inner surface. No Cu was seen on the outer surface that had been previously contacted with Teflon. (Bottom) Enlarged image of the copper film (0.8 mm × 0.6 mm) where features such as lines reflect the surface structure of the tube instead of scratches. (Upper right) Images of two glass test tubes containing copper plating solution before and after the plating. The blue color of the solution disappeared following the deposition (reprinted with permission from Ref. [95]. 2009, American Chemical Society).

The electron transfer during S–L CE initiated the generation of free radicals and various reactive oxygen species, enabling the catalytic degradation of organic pollutants. Wang et al. [52] demonstrated that the electrons transferred between pristine dielectric powder and water via S–L CE could directly catalyze reactions, unlike conventional catalytic processes that typically require external catalysts to drive the reaction. They investigated the degradation of methyl orange using CEC. The experimental setup is schematically illustrated in Figure 11a. In their study, FEP powder was added to an aqueous solution and stirred for 48 h to enhance the contact between FEP and water. Subsequently, the suspension was subjected to ultrasonication for 180 min. This process transformed the original yellow solution into a transparent one. The frequent CE at the FEP–water interface facilitated electron exchange, thereby generating reactive oxygen species, which effectively degraded the methyl orange in the aqueous solution. In addition, Wang et al. [52] proposed a mechanism for the degradation of methyl orange via free radicals generated during CEC, as depicted in Figure 11b. The proposed degradation pathway of organic pollutants through CEC involves two concurrent processes. First, electron transfer between water and FEP during the CE process generates radical cations. These radical cations rapidly undergo proton transfer from water, leading to the formation of hydrated hydronium ions and ·OH radicals. Second, the electrons accumulated on the FEP surface are captured by dissolved oxygen (O_2_) to form ·O_2_^−^ radicals. Subsequently, ·O_2_^−^ radicals react with protons to yield HO_2_·, which undergoes a chain reaction to produce additional ·OH radicals. These ·OH radicals interact with organic pollutants in the aqueous solution, driving the degradation of organic matter. Furthermore, the study highlighted the reduction of metals as critical evidence of electron transfer during CEC, corroborating the electron-driven mechanism of pollutant degradation.

Su et al. [54] successfully initiated reduction reactions under ultrasonic conditions using the CEC mechanism. Their study focused on the CE of noble metal ions in aqueous solutions, demonstrating that ultrasonic CEC effectively drove the reduction of metal ions such as Ag(ac), Rh^3^^+^, [PtCl_4_]^2^^−^, Ag^+^, Hg^2⁺^, Pd^2⁺^, [AuCl_4_]^−^, and Ir^3⁺^ [54]. Figure 11c presents a schematic of the experimental setup for the reduction of metal ions via sonication in the presence of FEP. A specific amount of dielectric insulator powder was introduced into a beaker containing a metal ion solution and sonicated for a predetermined duration. To minimize the influence of oxygen, the experiments were conducted under anaerobic conditions. Within minutes of sonication, noticeable color changes in the solution and precipitate formation were observed. Figure 11d illustrates the ultrasound-driven reduction of metal ions by CE in aqueous solutions facilitated by FEP. Notably, this aqueous–solid CE-based approach demonstrated its ability to extract gold from synthetic solutions at concentrations ranging from as low as 0.196 ppm to 196 ppm. This catalytic principle significantly enhanced catalytic efficiency, providing a promising pathway for efficient metal ion reduction and recovery. Similar results were observed by Liu et al. [95], as depicted in Figure 11e, where pristine PMMA demonstrated the spontaneous ability to transfer electrons into liquids, thereby inducing various electron transfer reactions. Their experiments revealed that Ag^+^, Cu^2⁺^, and Pd^2⁺^ ions were reduced and deposited onto the PMMA surface, while Fe(CN)_6_^3^^−^ was reduced to Fe(CN)_6_^4^^−^. Additionally, when PMMA powder was introduced into a slightly acidic solution, an increase in pH and the generation of hydrogen gas were observed. Chemiluminescence was also produced in a solution containing Ru(bpy)_3_^2⁺^ and S_2_O_8_^2^^−^ upon the addition of PMMA powder. These findings confirmed that PMMA becomes electrically charged upon contact with water, facilitating the participation of transferred electrons in redox reactions.

While a series of catalytic reactions initiated by CE were described above, the mechanism of how CE affects and regulates chemical reactions remains unclear. Wei et al. [55] introduced a CE-Chemistry paradigm involving a solid dielectric and a liquid, demonstrating that their physical contact induces and drives redox trends in chemical reactions. Notably, this study marked the first application of CE-Chemistry in non-aqueous systems, further underscoring the universality of its underlying mechanism. Additionally, Wei et al. [96] extended CE-Chemistry to organic systems for the first time, further broadening its scope. It established a groundbreaking framework for CE-Chemistry in nonaqueous systems, with Dimethyl Sulfoxide (DMSO) emerging as a particularly advantageous and unique solvent. Unlike aqueous systems, certain organic solvents, including DMSO, avoid ion generation during CE, effectively mitigating the screening effects of the EDL. Phenol degradation in DMSO showcased an unprecedented 30-fold faster reaction rate compared to deionized water, where complete degradation remains unattainable. Moreover, CE-driven phenol degradation in DMSO surpassed traditional mechano-driven methods, achieving a reaction rate over 40 times higher. It highlights the superior efficiency, chemoselectivity, and mechanoluminescence properties of DMSO-based systems, establishing a new paradigm in CE-Chemistry for nonmetal catalysis and providing deep insights into reaction kinetics and mechanisms.

**Figure 12:**
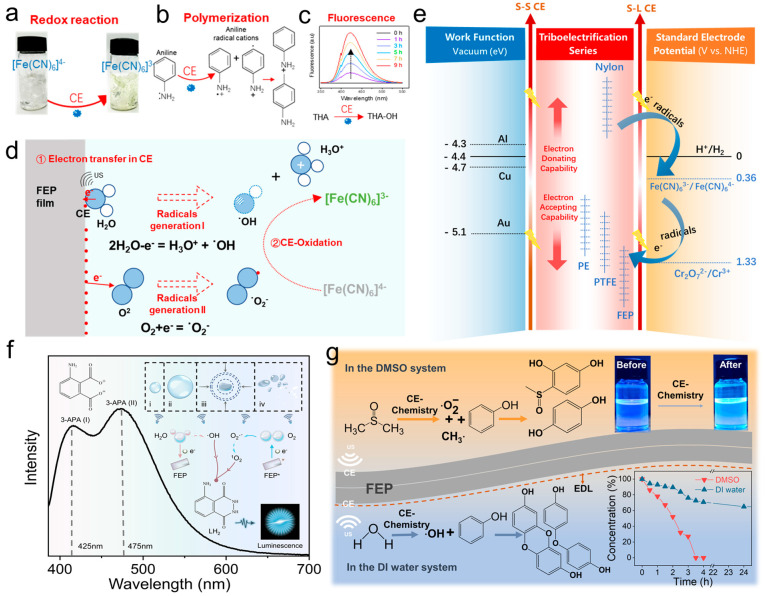
A comprehensive paradigm of CE-Chemistry reactions. (**a**) Schematic diagram of Fe^2+^ being oxidized to Fe^3+^. (**b**) Schematic diagram of the structure of aniline polymerization. (**c**) UV–vis spectra of solution samples at different times. (**d**) Working mechanism of CE oxidation reaction of Fe(CN)_6_^4−^ to Fe(CN)_6_^3−^ (reprinted with permission from Ref. [55]. 2024, Elsevier). (**e**) Guidance that unified the concept of work functions, triboelectric series, and standard electrode potentials by electron transfer capability. (**f**) Complete schematic of luminol CEL. The processes (**i**–**iv**) upper right corner of the figure represent the formation, growth, and collapse of cavitation bubbles provoked by the propagation of ultrasonic waves in solution (reprinted with permission from Ref. [31]. 2024, Elsevier). (**g**) Schematic diagram of the chemical reaction for the degradation of phenol in DMSO by CE (reprinted with permission from Ref. [97]. 2024, American Chemical Society).

Unlike chemical reactions triggered by light, heat, electricity, etc., CE-Chemistry based on the mechanical contact separation method opened up a new way to study the interfacial interactions among physics, materials science, and chemistry. As shown in Figure 12a, Fe(CN)_6_^4−^ was oxidized to Fe(CN)_6_^3−^ by S–L CE. A schematic structure of aniline polymerization was shown in Figure 12b, where different forms of aniline radical cations are induced by CE, two of which might react in a head-to-tail fashion to form intermediates or AOs for further polymerization. Figure 12c shows the UV–vis spectra of the solution samples at different times, from which it could be seen that the absorption peaks gradually increased as the reaction time increased. Figure 12d shows the schematic diagram of the reaction at the FEP–water interface. On the one hand, electrons would be transferred from the water molecules to the FEP–water interface to produce HO_2_^+^ and ·OH. On the other hand, both radicals were oxidative in nature, and O_2_ would obtain electrons from the FEP–water interface to produce ·O_2_^−^ and oxidize Fe^2+^ to Fe^3+^ via CE oxidation. As shown in Figure 12e [55], the correlation between electron negativity, work function, and electron chemistry redox standard potential of solid dielectrics (Al, Cu, Au) in the friction electric series was connected by CE. CE-Chemistry was demonstrated to be not only a physical process but also a physicochemical process, including charge transfer, free radical generation, a series of subsequent chemical reactions, and a unified figure of merit. A friction electrode series was formulated (a number of inert dielectrics are listed, such as Teflon, FEP, or PE, which showed the advantage of the wide choice of materials for CE-Chemistry and standard electrode potentials) as well as a guide to standard electrode potential concepts such as the reduction of H_2_, Fe^2+^, etc. This provided a new approach to guiding chemical reactions but also offered new perspectives for the development of multidisciplinary disciplines in physics, materials science, and chemistry.

Wei et al. [31] proposed a simple but effective approach to directly oxidize luminol via reactive oxygen species generated through CE between inert solid dielectrics and deionized water, termed contact electro-luminescence (CEL). This strategy employs metal-free, cost-effective solid dielectrics as a sustainable alternative to expensive metal-based catalysts, aligning with the principles of green chemistry and sustainable development. In this reaction system, the production of reactive oxygen radicals could be modulated by various solid dielectrics and additives, such as xylitol [97] and p-benzoquinone [98], thereby enabling precise tuning of the luminol reaction. Notably, two distinct luminescent emission peaks were directly observed, and their intensities, corresponding to the intermediate states, could be regulated (Figure 12f). The identification of these intermediates provided valuable insights into the intrinsic mechanisms of luminescence reactions, facilitating the development of effective and tunable luminescent systems. Furthermore, CEL introduces a novel mechanical–photonic conversion paradigm by harnessing triboelectric charge from the CE effect to study luminol luminescence. This method offers unique characteristic signals with significant potential for high efficiency and specific applications in environmental monitoring, biomedical diagnostics, and food safety testing. Recent advancements in leveraging triboelectric charge from S–L CE have highlighted its transformative potential in enhancing reaction efficiency. However, its application in aqueous solutions remains constrained by interfacial EDL screening. Wei et al. [96] further addressed this limitation by conducting CE-driven chemistry in nonaqueous systems, demonstrating its profound implications for catalysis and luminescence (Figure 12g). Through a combination of density functional theory simulations and experimental investigations, the study revealed significant variations in electron transfer efficiency and chemoselectivity across different solvents. Luminol oxidation in DMSO, catalyzed solely by radicals generated via CE, eliminated reliance on traditional catalysts and avoided side reactions, resulting in a pure, simplified system with stable luminescence maintained for over three months, significantly enhancing imaging accuracy and stability. It underscores the potential of triboelectric charge in transforming reaction kinetics and chemoselectivity, establishing a new paradigm in nonmetal catalysis and mechanoluminescence. These findings offer critical insights into reaction mechanisms and pave the way for advancements in green chemistry and sustainable technologies.

### 4.3. Alternative Approaches to S–L CE

**Figure 13:**
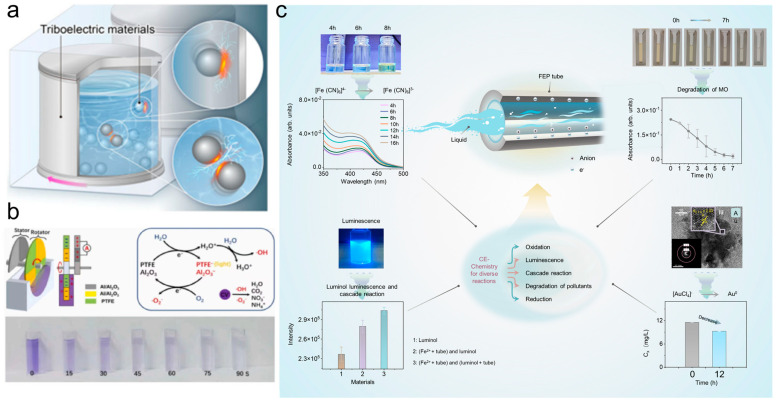
Other ways in which exposure electrocatalysis induces chemical reactions. (**a**) Schematic illustration of a ball mill process using triboelectric materials (reprinted with permission from Ref. [101]. 2024, Springer). (**b**) The designed catalysis unit including stator and rotator. The production process of ·OH and ·O_2_ and final degradation products of CV molecules. Optical photographs of 20 mg L^−1^ CV solution samples from 0 s to 90 s (reprinted with permission from Ref. [102]. 2023, Elsevier). (**c**) CE-Chemistry for K_4_[Fe(CN)_6_] solution and FEP tubes (reprinted with permission from Ref. [31]. 2024, Elsevier).

Ultrasound enhances CE through the combined effects of mechanical action, interfacial activation, and electron transfer [99,100], with its influence varying across materials. The mechanical effects of ultrasound, such as inducing vibration and displacement, play a critical role in these processes. At the S–L interface, ultrasound-induced frictional motion between water and the solid surface generates positive charges on water droplet surfaces. This frictional motion arises from ultrasonic mechanical vibrations, which induce relative movement between liquid molecules and the solid surface, thereby promoting charge transfer. Beyond its fundamental role, ultrasound has become a widely employed mechanical stimulus for achieving CE-Chemical reactions, underscoring its significance in advancing CE applications. Wang et al. [101] demonstrated a CEC process for reactive oxygen species generation via ball milling using triboelectric materials. Their study established that ball milling could achieve CEC through inert and conventional triboelectric materials. Experiments focused on the generation of reactive oxygen species during ball milling in a system where the triboelectric material constituted the milling vessel. The schematic of the ball milling process utilizing triboelectric materials is depicted in Figure 13a. The vial and ball, made from triboelectric material, resemble the setup of traditional liquid-assisted grinding (LAG). The frequent collisions occurring naturally during milling were hypothesized to induce significant CE phenomena. These CE-driven interfacial electron transfers were posited to enhance reaction rates, thereby enabling CEC. It was suggested that mechanical collision not only maximized the overlap in electron wave functions across the interface but also excited phonons that provide the energy for electron transition.

Zhang et al. [102] introduced a wind-driven, Al@Al_2_O_3_ (aluminum core–alumina shell construction material) and Teflon-based rotational S–L TENG device, designed for CEC and adsorption (Figure 13b). By leveraging CE at the S–L interface during mechanical rotation, the device significantly outperformed conventional advanced oxidation processes in treating organic pollutants. Using crystal violet (CV) as a model pollutant, the device achieved kinetic rate constants of 2.24 min^−1^ for low-concentration CV and 0.26 min^−1^ for high-concentration CV at a linear rotor speed of 1.41 m·s^−1^, representing a remarkable breakthrough in catalytic efficiency. Moreover, the system incorporated real-time monitoring capabilities for tracking the degradation progress of organic pollutants, further enhancing its practicality and innovation in catalytic applications. Moreover, Wei et al. [103] presented a versatile and recyclable CE-Chemistry approach, leveraging triboelectric charge generated by flow electrification (FE) to directly facilitate chemical reactions, including [Fe(CN)_6_]^4^^−^ oxidation, [AuCl_4_]^−^ reduction, and luminol luminescence (Figure 13c). Reaction rates were tunable through the adjustment of physical parameters of dielectric tubes, such as input flow velocity, inner diameter, length, and series-parallel configurations. Furthermore, cascade reactions, inspired by Flow Injection Analysis (FIA) systems in analytical chemistry, were successfully implemented. The Fe-induced CE-Chemistry in dielectric tubes offers promising applications in the real-time catalytic degradation of organic waste and the reduction of precious metals in wastewater systems [90,102,104,105,106]. This paradigm also calls for a reevaluation of traditional flow-based analytical methods, such as high-performance liquid chromatography (HPLC), in light of its potential catalytic functionalities. CE-Chemistry induced by S–L CE presented a transformative avenue for controlling and enhancing reaction kinetics at the interface, offering significant promise for next-generation chemical processes. This mechanism could drive innovations in green chemistry, wastewater treatment, and energy conversion, paving the way for more efficient and sustainable industrial applications.

## 5. Conclusions

**Figure 14:**
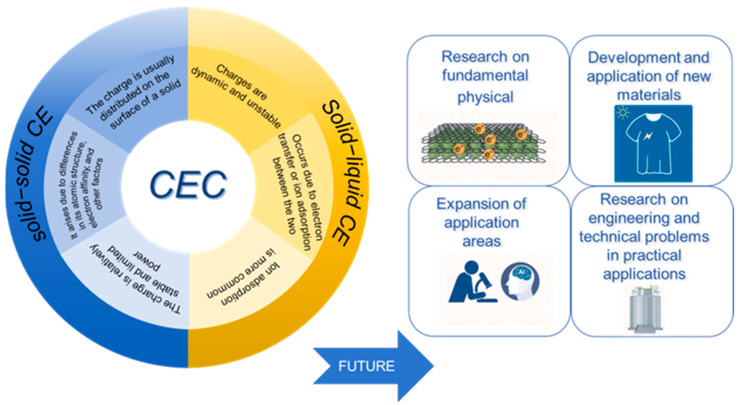
Characteristics of CE and future development direction.

In conclusion, this work compared the key properties of S–S and S–L CE, highlighting their differences and similarities in reaction rate, interfacial dynamics, and other aspects, while summarizing their respective merits and demerits to provide a clear reference framework for future research (Table 1). As an emerging CE-driven reaction mechanism, CE-Chemistry exemplifies the transformative potential of CE in initiating and regulating chemical reactions. As seen in Figure 14, both S–S and S–L CE provide diverse pathways and control strategies, enabling unique charge transfer modes by tailoring material properties, interfacial structures, and operational conditions. This approach eliminates the need for external power sources, allowing for chemical reactions to proceed solely through interfacial interactions, marking a significant advancement in sustainable and efficient reaction methodologies. S–S CE-Chemical reactions, while exhibiting relatively lower charge transfer efficiency compared to S–L systems, benefit from higher controllability and long-term stability. By optimizing parameters such as contact frequency, surface microstructures, friction coefficients, and environmental conditions (e.g., humidity, temperature), the efficiency of S–S CE-Chemistry can be significantly improved. Surface modifications, including the incorporation of micro/nanostructures or electronegative functional groups, further enhance interfacial charge density and stability, broadening its applicability in interfacial chemistry. In contrast, S–L CE-Chemistry systems excel in charge transfer efficiency, offering unique advantages in catalysis. However, the EDL screening effect in liquid-phase environments presents challenges, such as hindered charge transfer and reduced reaction stability. Strategies to overcome these limitations include adjusting solution pH, enhancing material surface roughness, or replacing aqueous media with organic solvents, which mitigate EDL screening effects and improve reaction efficiency. These advancements are particularly beneficial for rapid charge transfer in electrochemical reactions and energy devices. Future research should focus on the synergistic interactions between S–S and S–L CE-Chemical reactions, as their coexistence in multiphase systems could lead to coupling effects through charge compensation or interfacial electron transfer. Such interactions may enhance charge transfer efficiency and accelerate reaction rates, offering new insights into the fundamental mechanisms of CE-Chemistry. Understanding these synergies holds significant potential for breakthroughs in interdisciplinary applications, paving the way for innovative technologies in sustainable chemistry. Moreover, the potential of CE-Chemistry transcends optimizing charge transfer and efficiency, opening avenues for integration with emerging technologies. By leveraging nanomaterials, intelligent interfaces, and artificial intelligence (AI), CE-Chemistry can achieve precise monitoring, parameter optimization, and efficiency prediction. For instance, AI algorithms could adaptively optimize the complex parameters governing CE-Chemistry reactions, enabling real-time control and enhancing the efficiency of CE-Chemistry systems. Additionally, the molecular-level design of advanced materials and highly electronegative, durable interfacial coatings will significantly improve the stability and longevity of CE-Chemistry reactions, making them ideal for long-term applications. Future research will not only deepen CE-Chemistry’s role in fundamental science but also cement its impact on environmental remediation, next-generation energy conversion, and low-carbon manufacturing. As CE converges with cutting-edge advancements, CE-Chemistry is poised for broader applications, particularly in sustainable technologies, offering robust support for the global green transition.

## Figures and Tables

**Table 1:** The comparison between S–S and S–L CE.

Property	S–S CE	S–L CE
Charge Transfer Efficiency	Moderate to High, depends on surface interaction	High, influenced by solvent and interfacial properties
Reaction Rate	Faster in dry conditions, limited by surface area	Can be enhanced by solvent properties and humidity
Interfacial Dynamics	Primarily surface charge transfer, limited by surface roughness and contact force	Affected by solvent composition, ionic mobility, and surface wettability
Material Requirements	Requires high surface quality, minimal contamination	Requires compatible solvents for optimal performance
Merits	Simple system, potential for robust materials	High control over reaction rates, adaptable to various environments
Demerits	Limited scalability, sensitive to surface degradation	Dependent on solvent stability, possible side reactions with solvent
Applications	Triboelectric energy harvesting, sensors, coatings	catalytic reactions, green chemistry
Challenges	Surface degradation, difficulty in controlling charge transfer	Solvent-induced variations, material compatibility issues

## Data Availability

No new data were created or analyzed in this study.

## References

[1] Hansen L., Wollmann A., Weers M., Benker B., Weber A.P. (2020). Triboelectric Charging and Separation of Fine Powder Mixtures. Chem. Eng. Technol..

[2] Zhang W., Shi Y., Li Y., Chen X., Shen H. (2022). A Review: Contact Electrification on Special Interfaces. Front. Mater..

[3] Xu Y., Wu S., Zhu Y., Wu J. (2024). An Adhesion Model for Contact Electrification. Int. J. Mech. Sci..

[4] Li H., Fang X., Li R., Liu B., Tang H., Ding X., Xie Y., Zhou R., Zhou G., Tang Y. (2020). All-Printed Soft Triboelectric Nanogenerator for Energy Harvesting and Tactile Sensing. Nano Energy.

[5] Mannerbro R., Ranlöf M., Robinson N., Forchheimer R. (2008). Inkjet Printed Electrochemical Organic Electronics. Synth. Met..

[6] Huang S., Yao W., Hu J., Xu X. (2015). Tribological Performance and Lubrication Mechanism of Contact-Charged Electrostatic Spray Lubrication Technique. Tribol. Lett..

[7] Su Y., Zhang D., Wang B., Chu Z., Liu Z. (2022). Numerical Analysis of Droplet Transport Process in Composite Electrostatic Spraying (CES) Milling. J. Manuf. Process..

[8] Pan S., Zhang Z. (2019). Fundamental Theories and Basic Principles of Triboelectric Effect: A Review. Friction.

[9] Mitolo M., Di Lorenzo G., Stracqualursi E., Araneo R. (2024). Electrostatic Hazards in Power Systems. Proceedings of the 2024 IEEE International Conference on Environment and Electrical Engineering and 2024 IEEE Industrial and Commercial Power Systems Europe (EEEIC/I&CPS Europe).

[10] Michael J.B. (2019). Assessing the Trustworthiness of Electronic Systems. Computer.

[11] Niu Y., Li Z., Kong B., Wang E., Lou Q., Qiu L., Kong X., Wang J., Dong M., Li B. (2018). Similar Simulation Study on the Characteristics of the Electric Potential Response to Coal Mining. J. Geophys. Eng..

[12] Glor M. (2005). Electrostatic Ignition Hazards in the Process Industry. J. Electrost..

[13] Cai C., Luo B., Liu Y., Fu Q., Liu T., Wang S., Nie S. (2022). Advanced Triboelectric Materials for Liquid Energy Harvesting and Emerging Application. Mater. Today.

[14] Qian H., Peng P., Fan H., Yang Z., Yang L., Zhou Y., Tan D., Yang F., Willatzen M., Amaratunga G. (2024). Horizontal Transport in Ti_3_ C_2_ T_x_ MXene for Highly Efficient Osmotic Energy Conversion from Saline-Alkali Environments. Angew. Chem. Int. Ed..

[15] Wang Z.L. (2013). Triboelectric Nanogenerators as New Energy Technology for Self-Powered Systems and as Active Mechanical and Chemical Sensors. ACS Nano.

[16] Duque M., Murillo G. (2022). Tapping-Actuated Triboelectric Nanogenerator with Surface Charge Density Optimization for Human Motion Energy Harvesting. Nanomaterials.

[17] Mohamadbeigi N., Shooshtari L., Fardindoost S., Vafaiee M., Iraji Zad A., Mohammadpour R. (2024). Self-Powered Triboelectric Nanogenerator Sensor for Detecting Humidity Level and Monitoring Ethanol Variation in a Simulated Exhalation Environment. Sci. Rep..

[18] Li S., Liu J., Wang Z.L., Wei D. (2024). Mechano-Driven Chemical Reactions. Green. Energy Environ..

[19] Mi Q., Dong Y., Ge D., Xie S., Tian Y., Zou F., Yu H.-Y., Tam K.C. (2024). Scalable Manufacture of Efficient, Highly Stable, and Compact 3D Imitation Skin-Based Elastic Triboelectric Nanogenerator for Energy Harvesting and Self-Powered Sensing. Nano Energy.

[20] Barkas D.A., Psomopoulos C.S., Papageorgas P., Kalkanis K., Piromalis D., Mouratidis A. (2019). Sustainable Energy Harvesting through Triboelectric Nano—Generators: A Review of Current Status and Applications. Energy Procedia.

[21] Wang Y., Jin X., Wang W., Niu J., Zhu Z., Lin T. (2021). Efficient Triboelectric Nanogenerator (TENG) Output Management for Improving Charge Density and Reducing Charge Loss. ACS Appl. Electron. Mater..

[22] Zhang Q., Jin T., Cai J., Xu L., He T., Wang T., Tian Y., Li L., Peng Y., Lee C. (2022). Wearable Triboelectric Sensors Enabled Gait Analysis and Waist Motion Capture for IoT-Based Smart Healthcare Applications. Adv. Sci..

[23] Liu H., Dong J., Zhou H., Yang X., Xu C., Yao Y., Zhou G., Zhang S., Song Q. (2021). Real-Time Acid Rain Sensor Based on a Triboelectric Nanogenerator Made of a PTFE–PDMS Composite Film. ACS Appl. Electron. Mater..

[24] Hassan Q., Viktor P.J., Al-Musawi T., Mahmood Ali B., Algburi S., Alzoubi H.M., Khudhair Al-Jiboory A., Zuhair Sameen A., Salman H.M., Jaszczur M. (2024). The Renewable Energy Role in the Global Energy Transformations. Renew. Energy Focus.

[25] Beretta D., Neophytou N., Hodges J.M., Kanatzidis M.G., Narducci D., Martin- Gonzalez M., Beekman M., Balke B., Cerretti G., Tremel W. (2019). Thermoelectrics: From History, a Window to the Future. Mater. Sci. Eng. R Rep..

[26] Zhao Z., Dai Y., Liu D., Zhou L., Li S., Wang Z.L., Wang J. (2020). Rationally Patterned Electrode of Direct-Current Triboelectric Nanogenerators for Ultrahigh Effective Surface Charge Density. Nat. Commun..

[27] Lacks D.J., Mohan Sankaran R. (2011). Contact Electrification of Insulating Materials. J. Phys. D Appl. Phys..

[28] Williams M.W. (2011). Triboelectric Charging of Insulators—Evidence for Electrons Versus Ions. IEEE Trans. Ind. Applicat..

[29] Harper W.R. (1951). The Volta Effect as a Cause of Static Electrification. Proc. R. Soc. Lond. A.

[30] Wang Z.L., Wang A.C. (2019). On the Origin of Contact-Electrification. Mater. Today.

[31] Xu C., Li S., Yang Z., Willatzen M., Lin Wang Z., Wei D. (2024). Contact-Electro-Luminescence Triggered by Triboelectric Charge. Chem. Eng. J..

[32] Ouyang Y., Li X., Li S., Wang Z.L., Wei D. (2024). Ionic Rectification by Dynamic Regulation of the Electric Double Layer at the Hydrogel Interface. ACS Appl. Mater. Interfaces.

[33] Zhang J., Coote M.L., Ciampi S. (2021). Electrostatics and Electrochemistry: Mechanism and Scope of Charge-Transfer Reactions on the Surface of Tribocharged Insulators. J. Am. Chem. Soc..

[34] Pandey R.K., Kakehashi H., Nakanishi H., Soh S. (2018). Correlating Material Transfer and Charge Transfer in Contact Electrification. J. Phys. Chem. C.

[35] Chen Z., Lu Y., Li R., Orlando R.J., Manica R., Liu Q. (2022). Liquid-Solid Triboelectric Nanogenerators for a Wide Operation Window Based on Slippery Lubricant-Infused Surfaces (SLIPS). Chem. Eng. J..

[36] McCarty L.S., Whitesides G.M. (2008). Electrostatic Charging Due to Separation of Ions at Interfaces: Contact Electrification of Ionic Electrets. Angew. Chem. Int. Ed..

[37] Grzybowski B.A., Fialkowski M., Wiles J.A. (2005). Kinetics of Contact Electrification between Metals and Polymers. J. Phys. Chem. B.

[38] Toulemonde M., Dufour C., Meftah A., Paumier E. (2000). Transient Thermal Processes in Heavy Ion Irradiation of Crystalline Inorganic Insulators. Nucl. Instrum. Methods Phys. Res. Sect. B Beam Interact. Mater. At..

[39] Song F., Wang Z., Ma T., Chen L., Li H., Wu F. (2023). Enhanced Electron Cloud through π-π Interaction in Charge-Transfer Complexes for All-Solid-State Lithium Batteries. Nano Energy.

[40] Ye C., Zhang D.-S., Chen B., Tung C.-H., Wu L.-Z. (2024). Interfacial Charge Transfer Regulates Photoredox Catalysis. ACS Cent. Sci..

[41] Baharfar M., Hillier A.C., Mao G. (2024). Charge-Transfer Complexes: Fundamentals and Advances in Catalysis, Sensing, and Optoelectronic Applications. Adv. Mater..

[42] Šutka A., Mālnieks K., Lapčinskis L., Timusk M., Kalniņš K., Kovaļovs A., Bitenieks J., Knite M., Stevens D., Grunlan J. (2020). Contact Electrification between Identical Polymers as the Basis for Triboelectric/Flexoelectric Materials. Phys. Chem. Chem. Phys..

[43] Xu C., Zhang B., Wang A.C., Zou H., Liu G., Ding W., Wu C., Ma M., Feng P., Lin Z. (2019). Contact-Electrification between Two Identical Materials: Curvature Effect. ACS Nano.

[44] Sobolev Y.I., Adamkiewicz W., Siek M., Grzybowski B.A. (2022). Charge Mosaics on Contact-Electrified Dielectrics Result from Polarity-Inverting Discharges. Nat. Phys..

[45] Baytekin H.T., Patashinski A.Z., Branicki M., Baytekin B., Soh S., Grzybowski B.A. (2011). The Mosaic of Surface Charge in Contact Electrification. Science.

[46] Nie J., Ren Z., Xu L., Lin S., Zhan F., Chen X., Wang Z.L. (2020). Probing Contact-Electrification-Induced Electron and Ion Transfers at a Liquid–Solid Interface. Adv. Mater..

[47] Qin C., Wang D., Liu Y., Yang P., Xie T., Huang L., Zou H., Li G., Wu Y. (2021). Tribo-Electrochemistry Induced Artificial Solid Electrolyte Interface by Self-Catalysis. Nat. Commun..

[48] Liu C., Bard A.J. (2010). Electrostatic Electrochemistry: Nylon and Polyethylene Systems. Chem. Phys. Lett..

[49] Melentiev R., Tao R., Fatta L., Tevtia A.K., Verghese N., Lubineau G. (2023). Towards Decoupling Chemical and Mechanical Adhesion at the Electroplated Metal/Polymer Interface via Precision Surface Texturing. Surf. Interfaces.

[50] Ochoa-Putman C., Vaidya U.K. (2011). Mechanisms of Interfacial Adhesion in Metal–Polymer Composites—Effect of Chemical Treatment. Compos. Part A Appl. Sci. Manuf..

[51] Melentiev R., Yudhanto A., Tao R., Vuchkov T., Lubineau G. (2022). Metallization of Polymers and Composites: State-of-the-Art Approaches. Mater. Des..

[52] Wang Z., Berbille A., Feng Y., Li S., Zhu L., Tang W., Wang Z.L. (2022). Contact-Electro-Catalysis for the Degradation of Organic Pollutants Using Pristine Dielectric Powders. Nat. Commun..

[53] Li X., Berbille A., Wang T., Zhao X., Li S., Su Y., Li H., Zhang G., Wang Z., Zhu L. (2024). Defect Passivation Toward Designing High-Performance Fluorinated Polymers for Liquid–Solid Contact-Electrification and Contact-Electro-Catalysis. Adv. Funct. Mater..

[54] Su Y., Berbille A., Li X.-F., Zhang J., PourhosseiniAsl M., Li H., Liu Z., Li S., Liu J., Zhu L. (2024). Reduction of Precious Metal Ions in Aqueous Solutions by Contact-Electro-Catalysis. Nat. Commun..

[55] Li S., Zhang Z., Peng P., Li X., Wang Z.L., Wei D. (2024). A Green Approach to Induce and Steer Chemical Reactions Using Inert Solid Dielectrics. Nano Energy.

[56] Xia X., Wang H., Guo H., Xu C., Zi Y. (2020). On the Material-Dependent Charge Transfer Mechanism Of The Contact Electrification. Nano Energy.

[57] Helseth L.E. (2019). The Influence of Microscale Surface Roughness on Water-Droplet Contact Electrification. Langmuir.

[58] Nie J., Wang Z., Ren Z., Li S., Chen X., Lin Wang Z. (2019). Power Generation from the Interaction of a Liquid Droplet and a Liquid Membrane. Nat. Commun..

[59] Xiao S., Wu H., Li N., Tan X., Deng H., Zhang X., Tang J., Li Y. (2023). Triboelectric Mechanism of Oil-Solid Interface Adopted for Self-Powered Insulating Oil Condition Monitoring. Adv. Sci..

[60] Yoo D., Kim S.J., Joung Y., Jang S., Choi D., Kim D.S. (2022). Lotus Leaf-Inspired Droplet-Based Electricity Generator with Low-Adhesive Superhydrophobicity for a Wide Operational Droplet Volume Range and Boosted Electricity Output. Nano Energy.

[61] Li W., Ma L., Xu X., Luo J. (2023). Bidirectional Electron Transfer in Triboelectrification Caused by Friction-Induced Change in Surface Electronic Structure. Nano Energy.

[62] Hu J., Iwamoto M., Chen X. (2024). A Review of Contact Electrification at Diversified Interfaces and Related Applications on Triboelectric Nanogenerator. Nano-Micro Lett..

[63] Xu C., Zi Y., Wang A.C., Zou H., Dai Y., He X., Wang P., Wang Y., Feng P., Li D. (2018). On the Electron-Transfer Mechanism in the Contact-Electrification Effect. Adv. Mater..

[64] Zhang C., Zhou L., Cheng P., Yin X., Liu D., Li X., Guo H., Wang Z.L., Wang J. (2020). Surface Charge Density of Triboelectric Nanogenerators: Theoretical Boundary and Optimization Methodology. Appl. Mater. Today.

[65] Bai P., Bazant M.Z. (2014). Charge Transfer Kinetics at the Solid–Solid Interface in Porous Electrodes. Nat. Commun..

[66] Senna M. (2001). Charge Transfer and Hetero-Bonding across the Solid–Solid Interface at Room Temperature. Mater. Sci. Eng. A.

[67] Wu X., Chen X., Zhang Q.M., Tan D.Q. (2022). Advanced Dielectric Polymers for Energy Storage. Energy Storage Mater..

[68] Diao C., Wang H., Wang B., He Y., Hou Y., Zheng H. (2022). Overviews of Dielectric Energy Storage Materials and Methods to Improve Energy Storage Density. J. Mater. Sci. Mater. Electron..

[69] Pattipaka S., Lim Y., Son Y.H., Bae Y.M., Peddigari M., Hwang G.-T. (2024). Ceramic-Based Dielectric Materials for Energy Storage Capacitor Applications. Materials.

[70] Nag A., Baksi A., Ghosh J., Kumar V., Bag S., Mondal B., Ahuja T., Pradeep T. (2019). Tribochemical Degradation of Polytetrafluoroethylene in Water and Generation of Nanoplastics. ACS Sustain. Chem. Eng..

[71] Liu C., Bard A.J. (2008). Electrostatic Electrochemistry at Insulators. Nat. Mater..

[72] Zhao J., Zhang X., Xu J., Tang W., Lin Wang Z., Ru Fan F. (2023). Contact-electro-catalysis for Direct Synthesis of H_2_O_2_ under Ambient Conditions. Angew. Chem. Int. Ed..

[73] Berbille A., Li X., Su Y., Li S., Zhao X., Zhu L., Wang Z.L. (2023). Mechanism for Generating H_2_O_2_ at Water-Solid Interface by Contact-Electrification. Adv. Mater..

[74] Liu C., Bard A.J. (2009). Electrons on Dielectrics and Contact Electrification. Chem. Phys. Lett..

[75] Baytekin B., Baytekin H.T., Grzybowski B.A. (2012). What Really Drives Chemical Reactions on Contact Charged Surfaces?. J. Am. Chem. Soc..

[76] Zhang J., Rogers F.J.M., Darwish N., Gonçales V.R., Vogel Y.B., Wang F., Gooding J.J., Peiris M., Chandramalika R., Jia G. (2019). Electrochemistry on Tribocharged Polymers Is Governed by the Stability of Surface Charges Rather than Charging Magnitude. J. Am. Chem. Soc..

[77] Suh I.-Y., Jeon J., Park M.J., Ryu H., Park Y.J., Kim S.-W. (2024). Recent Studies on Solid–Liquid Contact Electrification. ACS Appl. Electron. Mater..

[78] Byun K.-E., Cho Y., Seol M., Kim S., Kim S.-W., Shin H.-J., Park S., Hwang S. (2016). Control of Triboelectrification by Engineering Surface Dipole and Surface Electronic State. ACS Appl. Mater. Interfaces.

[79] Xu W., Zheng H., Liu Y., Zhou X., Zhang C., Song Y., Deng X., Leung M., Yang Z., Xu R.X. (2020). A Droplet-Based Electricity Generator with High Instantaneous Power Density. Nature.

[80] Kwak S.S., Lin S., Lee J.H., Ryu H., Kim T.Y., Zhong H., Chen H., Kim S.-W. (2016). Triboelectrification-Induced Large Electric Power Generation from a Single Moving Droplet on Graphene/Polytetrafluoroethylene. ACS Nano.

[81] EI-Kazzaz A., Rose-lnnes A.C. (1985). Contact charging of insulators by liquid metals. J. Electrost..

[82] Yatsuzuka K., Higashiyama Y., Asano K. (1994). Electrification of Polymer Surface Caused by Sliding Ultrapure Water. J. Electrost..

[83] Burgo T.A.L., Galembeck F., Pollack G.H. (2016). Where Is Water in the Triboelectric Series?. J. Electrost..

[84] Lin S., Xu L., Chi Wang A., Wang Z.L. (2020). Quantifying Electron-Transfer in Liquid-Solid Contact Electrification and the Formation of Electric Double-Layer. Nat. Commun..

[85] Lin S., Xu L., Zhu L., Chen X., Wang Z.L. (2019). Electron Transfer in Nanoscale Contact Electrification: Photon Excitation Effect. Adv. Mater..

[86] Willatzen M., Lew Yan Voon L.C., Wang Z.L. (2020). Quantum Theory of Contact Electrification for Fluids and Solids. Adv. Funct. Mater..

[87] Lin S., Chen X., Wang Z.L. (2022). Contact Electrification at the Liquid–Solid Interface. Chem. Rev..

[88] Loh Z.-H., Doumy G., Arnold C., Kjellsson L., Southworth S.H., Al Haddad A., Kumagai Y., Tu M.-F., Ho P.J., March A.M. (2020). Observation of the Fastest Chemical Processes in the Radiolysis of Water. Science.

[89] Gauduel Y., Pommeret S. (1990). Some Evidence of Ultrafast H_2_O^+^-Water Molecule Reaction in Femtosecond Photoionization of Pure Liquid Water: Influence on Geminate Pair Recombination Dynamics. Chem. Phys..

[90] Chen B., Xia Y., He R., Sang H., Zhang W., Li J., Chen L., Wang P., Guo S., Yin Y. (2022). Water–Solid Contact Electrification Causes Hydrogen Peroxide Production from Hydroxyl Radical Recombination in Sprayed Microdroplets. Proc. Natl. Acad. Sci. USA.

[91] Zhang J., Lin S., Wang Z.L. (2022). Electrostatic Charges Regulate Chemiluminescence by Electron Transfer at the Liquid–Solid Interface. J. Phys. Chem. B.

[92] Lui T.-Y., Chen X., Zhang S., Hu D., Dominic Chan T.-W. (2023). Peptide Oxidation Induced by Liquid–Solid Contact Electrification as Revealed in Liquid Microjunction-Surface Sampling Probe Mass Spectrometry. Anal. Chem..

[93] Zhao Y., Liu Y., Wang Y., Li S., Liu Y., Wang Z.L., Jiang P. (2023). The Process of Free Radical Generation in Contact Electrification at Solid-Liquid Interface. Nano Energy.

[94] Chen X., Liu Z., Wang Z. (2023). The process of interfacial electron transfer in liquid-solid contact and the two-step mechanism model of EDL structure. Sci. Sin. Tech..

[95] Liu C., Bard A.J. (2009). Chemical Redox Reactions Induced by Cryptoelectrons on a PMMA Surface. J. Am. Chem. Soc..

[96] Liu J., Yang Z., Li S., Du Y., Zhang Z., Shao J., Willatzen M., Wang Z.L., Wei D. (2024). Nonaqueous Contact-Electro-Chemistry via Triboelectric Charge. J. Am. Chem. Soc..

[97] Gao N., Ren G., Zhang M., Mao L. (2024). Electroless Deposition of Palladium Nanoparticles on Graphdiyne Boosts Electrochemiluminescence. J. Am. Chem. Soc..

[98] Peng Y., Yu L., Sheng M., Wang Q., Jin Z., Huang J., Yang X. (2023). Room-Temperature Synthesized Iron/Cobalt Metal–Organic Framework Nanosheets with Highly Efficient Catalytic Activity toward Luminol Chemiluminescence Reaction. Anal. Chem..

[99] Willatzen M., Wang Z.L. (2019). Contact Electrification by Quantum-Mechanical Tunneling. Research.

[100] Zhang D.L., Shi J.M., Wang Z.L., Tang W. (2022). Probing Polymer Contact Electrification by Gamma-Ray Radiation. Front. Mater..

[101] Wang Z., Dong X., Li X.-F., Feng Y., Li S., Tang W., Wang Z.L. (2024). A Contact-Electro-Catalysis Process for Producing Reactive Oxygen Species by Ball Milling of Triboelectric Materials. Nat. Commun..

[102] Zhang M., Song W.-Z., Chen T., Sun D.-J., Zhang D.-S., Li C.-L., Li R., Zhang J., Ramakrishna S., Long Y.-Z. (2023). Rotation-Mode Liquid-Solid Triboelectric Nanogenerator for Efficient Contact-Electro-Catalysis and Adsorption. Nano Energy.

[103] Xu C., Li S., Zhang Y., Wang Z., Wang Z.L., Wei D. (2025). Contact-Electro-Chemistry Induced by Flow Electrification in Dielectric Tubes. Nano Energy.

[104] Jiang B., Xue X., Mu Z., Zhang H., Li F., Liu K., Wang W., Zhang Y., Li W., Yang C. (2022). Contact-Piezoelectric Bi-Catalysis of an Electrospun ZnO@PVDF Composite Membrane for Dye Decomposition. Molecules.

[105] Chen Z., Lu Y., Liu X., Li J., Liu Q. (2023). Novel Magnetic Catalysts for Organic Pollutant Degradation via Contact Electro-Catalysis. Nano Energy.

[106] Zhang Y., Kang T., Han X., Yang W., Gong W., Li K., Guo Y. (2023). Molecular-Functionalized Metal-Organic Frameworks Enabling Contact-Electro-Catalytic Organic Decomposition. Nano Energy.

